# A novel Multi-Level Refined (MLR) knowledge graph design and chatbot system for healthcare applications

**DOI:** 10.1371/journal.pone.0296939

**Published:** 2024-01-31

**Authors:** Huei-Chia Hsueh, Shuo-Chen Chien, Chih-Wei Huang, Hsuan-Chia Yang, Usman Iqbal, Li-Fong Lin, Wen-Shan Jian

**Affiliations:** 1 Department of Pharmacy, Taipei Veterans General Hospital, Taipei, Taiwan; 2 Department of Artificial Intelligence in Medicine, Professional Master Program, Taipei Medical University, Taipei, Taiwan; 3 Graduate Institute of Biomedical Informatics, Taipei Medical University, Taipei, Taiwan; 4 International Research Center for Health Information Technology, School of Medical Science and Technology, Taipei Medical University, Taipei, Taiwan; 5 Department of Health, Health ICT, Tasmania, Australia; 6 Global Health and Health Security Department, College of Public Health, Taipei Medical University, Taipei, Taiwan; 7 School of Gerontology & Long-Term Care, Taipei Medical University, Taipei, Taiwan; 8 Department of Physical Medicine and Rehabilitation, Shuang Ho Hospital, Taipei Medical University, New Taipei, Taiwan; 9 Graduate Institute of Data Science, Taipei Medical University, Taipei, Taiwan; 10 School of Health Care Administration, Taipei Medical University, Taipei, Taiwan; The University of Lahore, PAKISTAN

## Abstract

Imagine having a knowledge graph that can extract medical health knowledge related to patient diagnosis solutions and treatments from thousands of research papers, distilled using machine learning techniques in healthcare applications. Medical doctors can quickly determine treatments and medications for urgent patients, while researchers can discover innovative treatments for existing and unknown diseases. This would be incredible! Our approach serves as an all-in-one solution, enabling users to employ a unified design methodology for creating their own knowledge graphs. Our rigorous validation process involves multiple stages of refinement, ensuring that the resulting answers are of the utmost professionalism and solidity, surpassing the capabilities of other solutions. However, building a high-quality knowledge graph from scratch, with complete triplets consisting of subject entities, relations, and object entities, is a complex and important task that requires a systematic approach. To address this, we have developed a comprehensive design flow for knowledge graph development and a high-quality entities database. We also developed knowledge distillation schemes that allow you to input a keyword (entity) and display all related entities and relations. Our proprietary methodology, multiple levels refinement (MLR), is a novel approach to constructing knowledge graphs and refining entities level-by-level. This ensures the generation of high-quality triplets and a readable knowledge graph through keyword searching. We have generated multiple knowledge graphs and developed a scheme to find the corresponding inputs and outputs of entity linking. Entities with multiple inputs and outputs are referred to as joints, and we have created a joint-version knowledge graph based on this. Additionally, we developed an interactive knowledge graph, providing a user-friendly environment for medical professionals to explore entities related to existing or unknown treatments/diseases. Finally, we have advanced knowledge distillation techniques.

## Introduction

Knowledge Graphs (KGs) were first introduced by Google in 2012 [1] with its general-purpose knowledge base. However, similar concepts have existed in the field of artificial intelligence for decades, including in areas such as knowledge representation, knowledge acquisition, natural language processing (NLP), ontology engineering, and the semantic web. Today, KGs are widely used in various applications such as search engines, chatbots, recommendation systems [2], and autonomous systems. Although KGs can be generated from unstructured data sources, the process of reading, processing, and filtering the information from multiple sources such as journals, papers, and online articles is time-consuming and challenging.

Our tool (MLR-KG) utilizes NLP and machine learning techniques to generate triplets from scratch for healthcare applications, specifically for the treatment of asthma. Unlike AI-KG (Artificial Intelligence Knowledge Graph) [3], it has been generated by applying an automatic pipeline that extracts entities and relationships using three tools: DyGIE++ [4], Stanford CoreNLP [5, 6], and the CSO Classifier [7]. On the contrast, we have developed our own unique solution that incorporates a multi-level refinement (MLR) process with professional validation to guarantee the quality of the generated triplets. This approach allows us to process large amounts of data and create a high-quality knowledge graph that can be used in various healthcare applications.

In addition to Neo4j, which can plot KGs and store the triplets in a graph database, we have our own approach to store and plot the KGs. We store the triplet data as ‘.csv’ files, which can be stored and rebuilt in databases such as Oracle. We use machine learning libraries to plot the Knowledge Graph (KG) and provide an interactive environment for researchers to explore and verify the entities. Furthermore, we have developed a joint-version KG that highlights the complex relationships between triplets using different colored nodes. Unlike Neo4j, our method allows for customization and visualization of joint nodes.

Initially, we attempted to use tools such as TextRazor, which is an API based tool that can be used from TextRazor’s website [8] or as a downloadable library, to identify entities, but setting up the environment to process multiple articles was challenging. We also explored existing tools for building triplets, but no existing tool could meet our requirements for building and customizing a KG in a one-stop shop. This was because of the need to consider refinement procedures during construction. To overcome these obstacles, we used Python to generate triplets by incorporating NLP techniques, plot basic, joint, and interactive KGs using the networkx python library, and distill final knowledge from the input keyword. This sequential design flow emphasized the use of Python instead of existing tools to develop our KGs, avoiding integration difficulties.

Our MLR-KG approach offers a complete solution for individuals or organizations looking to build their own KGs from scratch. This means that with MLR-KG, you can construct KGs, refine entities, explore entity relationships, and robustly distill knowledge and related entities and relations, all within the context of a specific search keyword. To demonstrate the effectiveness of MLR-KG, we selected articles related to asthma for a healthcare application. By using our approach, users can benefit from a step-by-step guide for building high-quality KGs, including the construction of entities, exploration of relationships, refinement, and distillation of knowledge. MLR-KG provides a novel and comprehensive methodology for KG development and is a valuable tool for those starting to construct their own KGs.

## Related work

In recent years, the construction of knowledge graphs (KGs) has attracted substantial attention, maintaining its status as an active and dynamic research field. Across diverse domains, a multitude of studies and innovative approaches have emerged, all aimed at constructing comprehensive KGs. In the subsequent discussion, we will delve into some pivotal works that have significantly influenced the landscape of KG construction. These examples not only illustrate the spectrum of methodologies and domains within this dynamic field but also underscore the multifaceted nature of KG research.

The highlighted approaches offer a rich variety of methods for KG construction and utilization. The triple-based approach [9], for instance, efficiently manages KGs in databases such as Apache Jena and Virtuoso, utilizing subject-predicate-object triples. Conversely, the Linked Data Approach [10] promotes data sharing by publishing RDF triples on the web, bolstered by unique URIs. Meanwhile, the NLP-based approach [11] plays a crucial role in extracting valuable KG data from unstructured text, extending its applications to domains like biomedicine and digital humanities.

In contrast, the Graph Embedding Approach [12] follows a distinct path, transforming KG entities and relationships into vectors suitable for tasks like link prediction and entity classification. In a comprehensive survey spanning seven knowledge domains [13], advanced techniques such as KG-BERT [14] and GraphSAGE for inductive node embeddings [15] are employed. This research delves into intricate aspects of human behavior [16], unraveling complex relationships between content and context through the use of an Attention-based LSTM [17]. Furthermore, the study illuminates the manifold advantages of KG embedding in downstream tasks [18].

Beyond these innovations, the author introduces a paraphrase detection method utilizing recursive autoencoders (RAE) and dynamic pooling, showcasing its superior performance on the Microsoft Research Paraphrase (MSRP) corpus [19]. The research boldly explores the domain of Graph Neural Networks (GNN), demonstrating their adaptability across various graph types in fields such as computer vision, chemistry, and data mining. Particularly, the utilization of TransE [20] for efficient entity and relationship embedding stands out, even excelling in demanding link prediction scenarios involving extensive datasets featuring millions of entities, tens of thousands of relationships, and millions of training samples [21]. This comprehensive exploration underscores the depth and breadth of contemporary KG research, highlighting its far-reaching implications in various domains.

However, the above methods did not match my goal to implement a professional, efficient, and high-quality knowledge graph (KG) with our proprietary design methodology. While these approaches had their merits, they fell short of meeting the specific requirements and standards we envisioned for our knowledge graph project. Our aim is to not only establish a robust and well-structured KG but also to ensure that it aligns seamlessly with our unique design philosophy and objectives. To achieve this, we have embarked on a distinct path that leverages our in-house expertise and tailored methodologies, allowing us to craft a KG that not only meets but surpasses the expectations we have set for its professionalism, efficiency, and overall quality.

The research on knowledge graphs in healthcare presented here stands as a substantial contribution to the field. It introduces a systematic, all-encompassing, and automated methodology for constructing professional knowledge graphs, proficiently categorizing data types and maintaining high data quality through rigorous validation by medical experts. The incorporation of an interactive framework for navigating complex relationships, along with the integration of knowledge distillation techniques, enhances the overall utility of the knowledge graph. The methodical step-by-step approach places a premium on entity quality throughout the process, ultimately yielding a comprehensive and user-friendly solution tailored for healthcare professionals and researchers.

## Methods

Our design flow is unique in that it utilizes multiple level refinement (MLR) techniques to refine the entities and relations in the triplets during the construction of the KG. This leads to the sequential creation of various Knowledge Graphs (KGs), encompassing a foundational KG, a joint-version KG, and an interactive KG, as illustrated in the design flow depicted in a design flow below.

**Fig 1:**
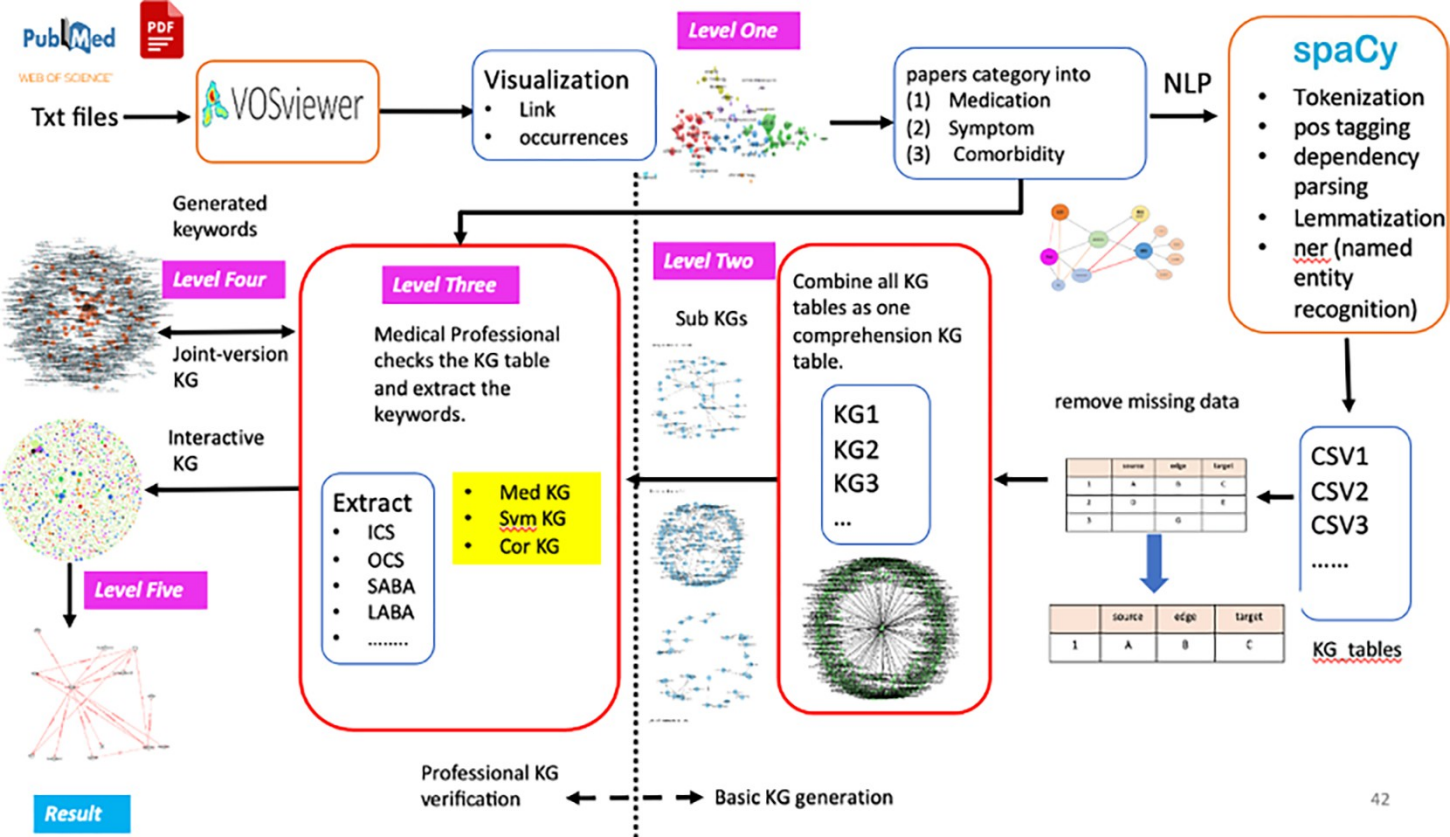
A design flow for Multi-Level Refined (MLR) KGs.

The MLR techniques are outlined in Fig 1. In Level One, we use VOSViewer to classify articles into different categories. For each category, we construct a corresponding KG. In Level Two, NLP techniques such as tokenization, tagging, part of speech, and named entity recognition are applied to each article. This results in a collection of triplets. These triplets are accumulated in a table and various KGs are constructed based on article categories. However, the initial KGs may contain a large amount of irrelevant information, making analysis challenging.

In Level Three, we have three KGs—Medication, Symptom, and Comorbidity. During the initial refinement process, we remove triplets with a single entity, a relation with only one entity, or two entities without a relation. We keep only complete triplets with two entities (subject and object) and one relation. The complete triplets are then stored in our database. Additionally, these entities are evaluated and validated by professional scholars, doctors, and researchers. Despite collecting these validated entities, the importance of each entity is still unknown. Hence, in Level Four, we create a joint-version KG, where the overlapping entities among the triplets are analyzed. We find many overlapping entities, and entities with more relations are considered more important. However, some entities with fewer relations might be more significant for specific applications. To explore entity relationships, we offer an interactive KG environment for researchers. Our goal is for researchers to discover both the most informative entities and the rarely crucial entities through this interactive KG design.

Finally, in Level Five, the knowledge distillation skills and plots are designed to extract desired entities from the KGs. The generated plot displays all connections of the selected entities. We are confident that there is no existing KG with the iterative capabilities of (i) generating KGs, (ii) refining entities through multiple levels, and (iii) exploring entity relationships all at once.

### 1. Data collection and article category

**Fig 2:**
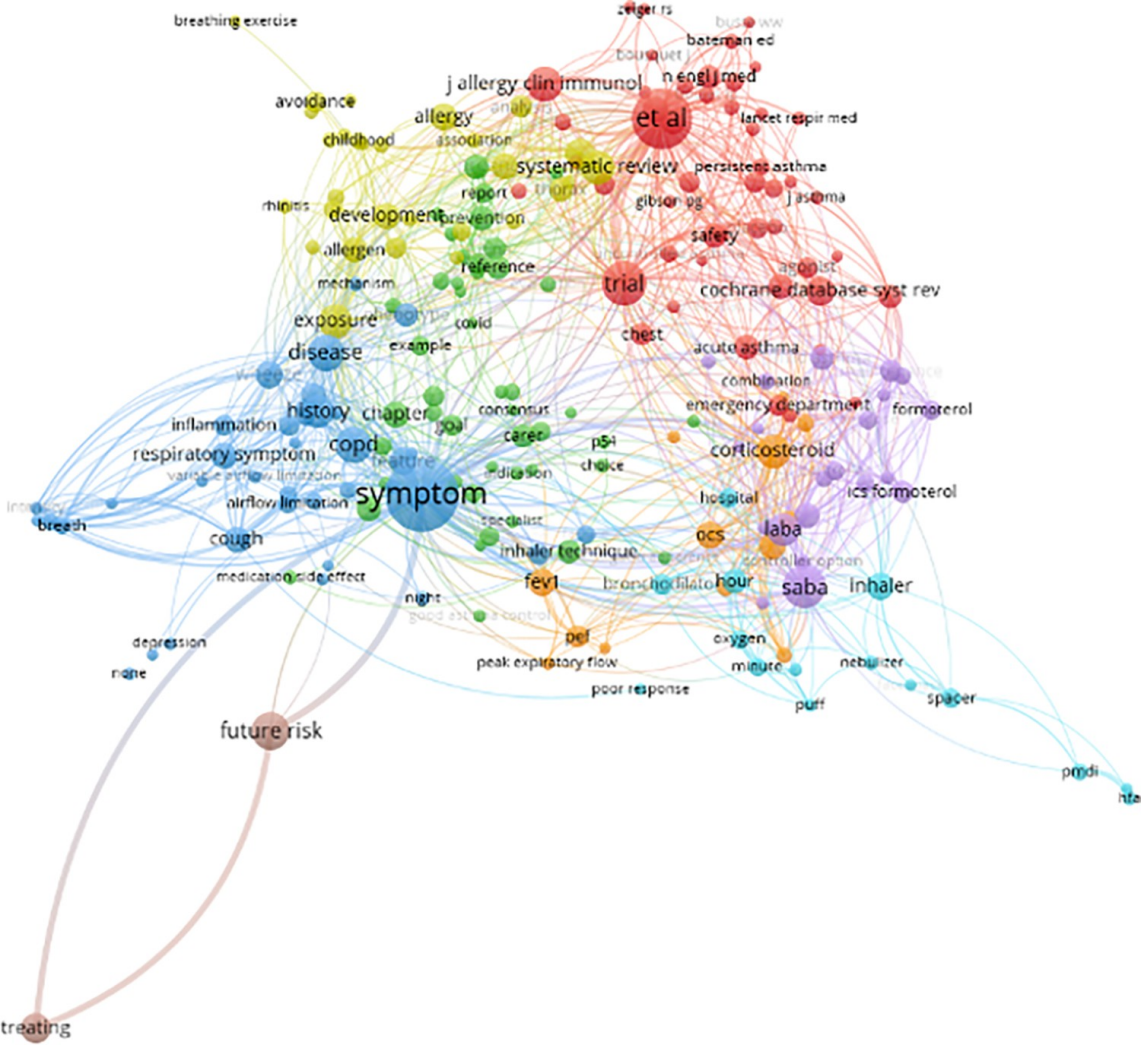
Co-occurrence analysis using VOSViewer of terms in GINA.

The data for the study is obtained from various medical literature websites including Google Scholar, PubMed, and the Web of Science (WOS) database. The articles selected are related to asthma and span from January 1, 2017 to July 31, 2021 and cover topics such as symptoms, treatment, diagnosis, triggers, comorbidity, medication, pediatrics, allergy, etc. Given the large volume of articles, manual reading and note-taking is not feasible. To overcome this challenge, machine learning is employed using VOSViewer to analyze the articles. The VOSViewer performs a co-occurrence cluster analysis, allowing for a preview of the articles. For instance, an article on the Global Initiative for Asthma (GINA) guideline can be assigned to three categories—symptoms (colored in blue), medication (colored in purple), and comorbidity (colored in yellow)—based on the major keywords identified by VOSViewer in Fig 2.

### 2. Basic KG construction

In our initial approach, we leveraged NLP technology, specifically Python SpaCy, to oversee text processing, entity recognition, and relation extraction. NLP techniques such as tokenization, tagging, part-of-speech analysis, and named entity recognition played pivotal roles in our article processing pipeline. These techniques generated triplets, comprising combinations of tokens, their associated parts of speech, and identified named entities. These triplets formed a well-structured foundation for a variety of NLP tasks, streamlining the extraction of insights and patterns from the text. To provide further clarity, our initial workflow initiated by segmenting all the text within a file into sentences, a process commonly known as tokenization. Each sentence was then disassembled into a subject, verb, and object, giving rise to what we frequently referred to as a triplet. In this context, the subject and object were representative of nouns, symbolizing entities, while the verbs conveyed the relationships between them. We reference Global Initiative for Asthma (GINA) [22] to leverage NLP processing.

Initially, we establish a fundamental KG utilizing an article titled ’GINA 2019’. Our Python code facilitates sentence tokenization from the article, generating a KG table that includes the subject, relation, and object for each sentence. The ultimate KG visualization is then created using the *networkx* Python library based on the information in the KG table. Below, you will find tables illustrating the outcomes of 9-sentence tokenization in Table 1 and 16 randomly selected triples in Table 2 that are specifically linked to the GINA article.

**Table 1:** Gina’s article in tokens: 9 selected sentences.

Index	Tokenized Sentence
605	Specific questions for assessment of asthma in children 6–11 years
2782	A trial of controller therapy should be given if the symptom pattern suggests asthma, alternative diagnoses have been excluded and respiratory symptoms are uncontrolled and/or wheezing episodes
1109	In severe asthma, as in mild-moderate asthma,245 participants in randomized controlled trials may not be representative of patients seen in clinical practice. For example, a registry study found
1253	FEV1: forced expiratory volume in 1 second; HDM: house dust mite; ICS: inhaled corticosteroids; OCS: oral corticosteroids; SLIT: sublingual immunotherapy.
1059	Based on product information, the maximum recommended dose of ICS-formoterol in a single day is a total of 48mcg formoterol for beclomethasone-formoterol, and 72mcg formoterol for budesonide-formoterol.
2519	‘Asthma-COPD overlap’ and ‘asthma +COPD’ are terms used to collectively describe patients who have persistent airflow limitation together with clinical features that are consistent with both.
679	taking controller treatment, or who has taken a short-acting beta2-agonist within 4 hours, or a LABA within 12 hours (or 24 hours for a once-daily LABA), suggests uncontrolled asthma.
320	Asthma is a heterogeneous disease, usually characterized by chronic airway inflammation. It is defined by the history of respiratory symptoms such as wheeze, shortness of breath, chest tightness.
1528	Food allergy as an exacerbating factor for asthma is uncommon and occurs primarily in young children. Confirmed food allergy is a risk factor for asthma-related mortality.

**Table 2:** Extracted triplets: 16 entries from knowledge graph.

Source	Edge	Target
ICS	included	SABA corticosteroid
Increase frequency	add	SABA spacer
acute care Patients	monitored	little SABA treatment
severe asthma	improve	SABA alone
ideally SABA reliever	Reducing	important asthma treatment
current GINA	recommends	serious dose ICS exacerbations
SABA	include	separate inhalers
Older inhaler patients	may	multiple inhaler devices
poor inhaler diagnosis	appear	incorrect inhaler technique
symptoms	relieved	inhaled bronchodilator
Oral bronchodilator therapy	recommended	inhaled SABA
Reslizumab benralizumab	binds to	cell eosinophils
Mechanism	signaling	interleukin-4 receptor IL-4
Clinician receptor judgment	used	worsening receptor asthma
eosinophilic airway patients	respond	corticosteroid asthma treatment
needed ICS formoterol	delivered	symptom relief

**Fig 3:**
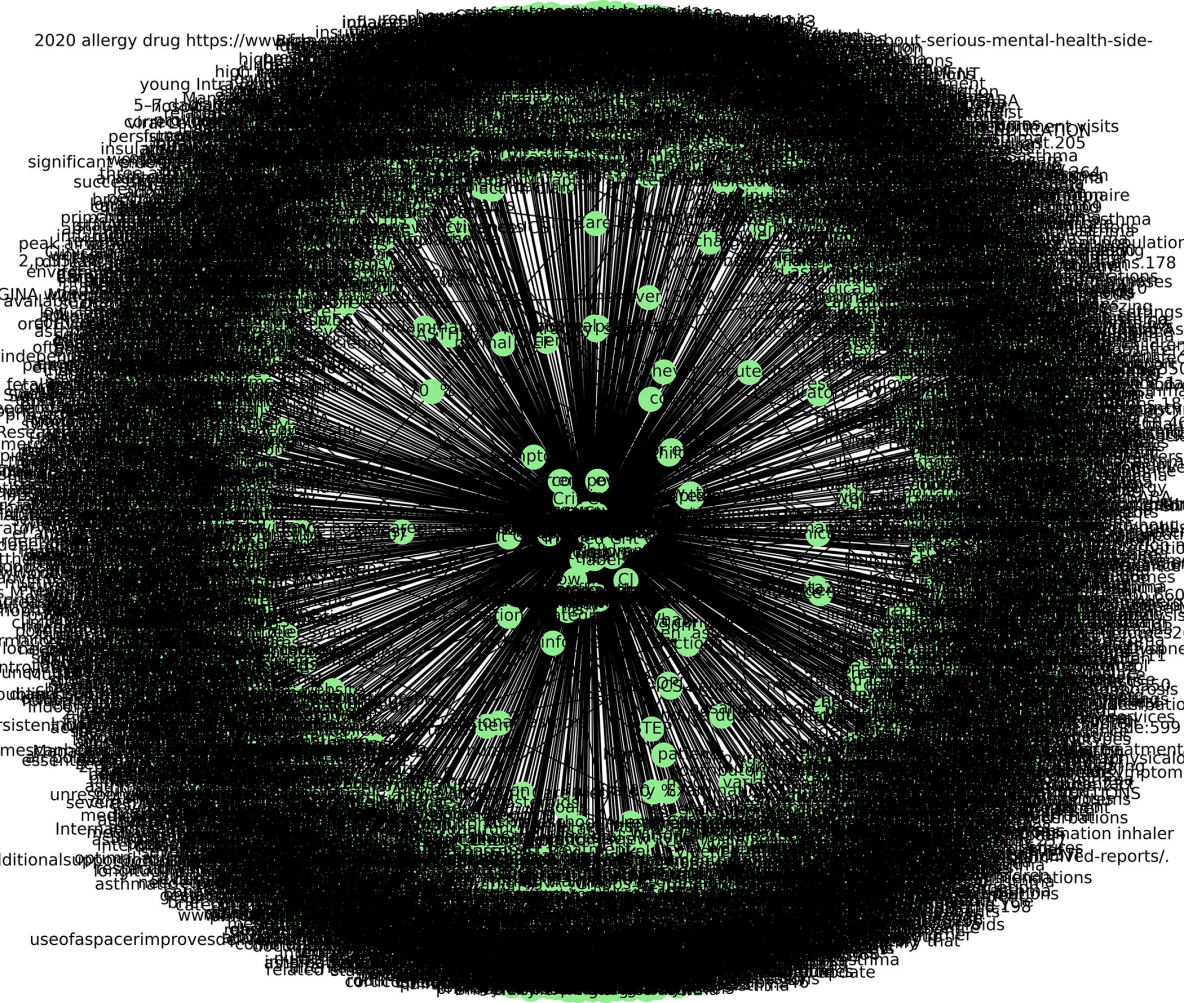
Building a knowledge graph using triples generated through NLP.

**Fig 4:**
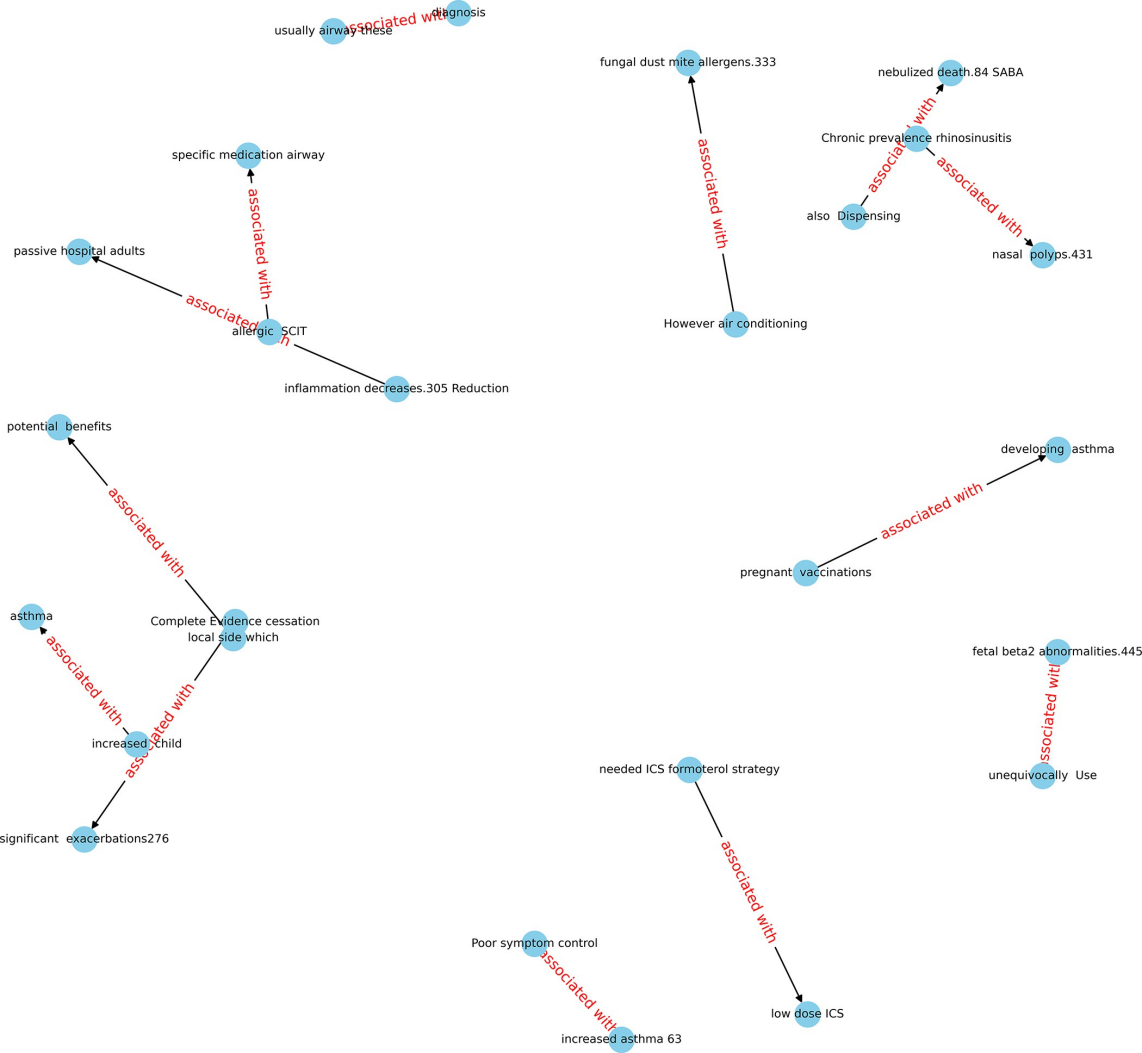
A sub-Knowledge Graph (sub-KG) is created using the relationship ’associated with’.

However, understanding the KG in Fig 3 is not straightforward. To get a better view, one may need to zoom into the graph to see its detailed information, known as a sub-KG. For instance, the relation "are" in displays the entities related to budesonide, symptoms, oral corticosteroids (OCS), and inhaled corticosteroids (ICS), and the relation "associated with" in Fig 4 displays entities related to short-acting beta agonists (SABA), airways, and asthma. These keywords are then added to our database (as seen in Fig 1, Level Two processing). However, there may be many irrelevant words that need to be removed and only the useful words kept in the triplet tables.

We have created three KGs based on three types of articles. For each graph, we have also developed several sub-graphs with the top 10 relations. Each relation sub-graph has numerous associated entities. These entities will be further validated by professional scholars and researchers through the process of Level Three processing.

### 3. Joint-version KG construction

**Fig 5:**
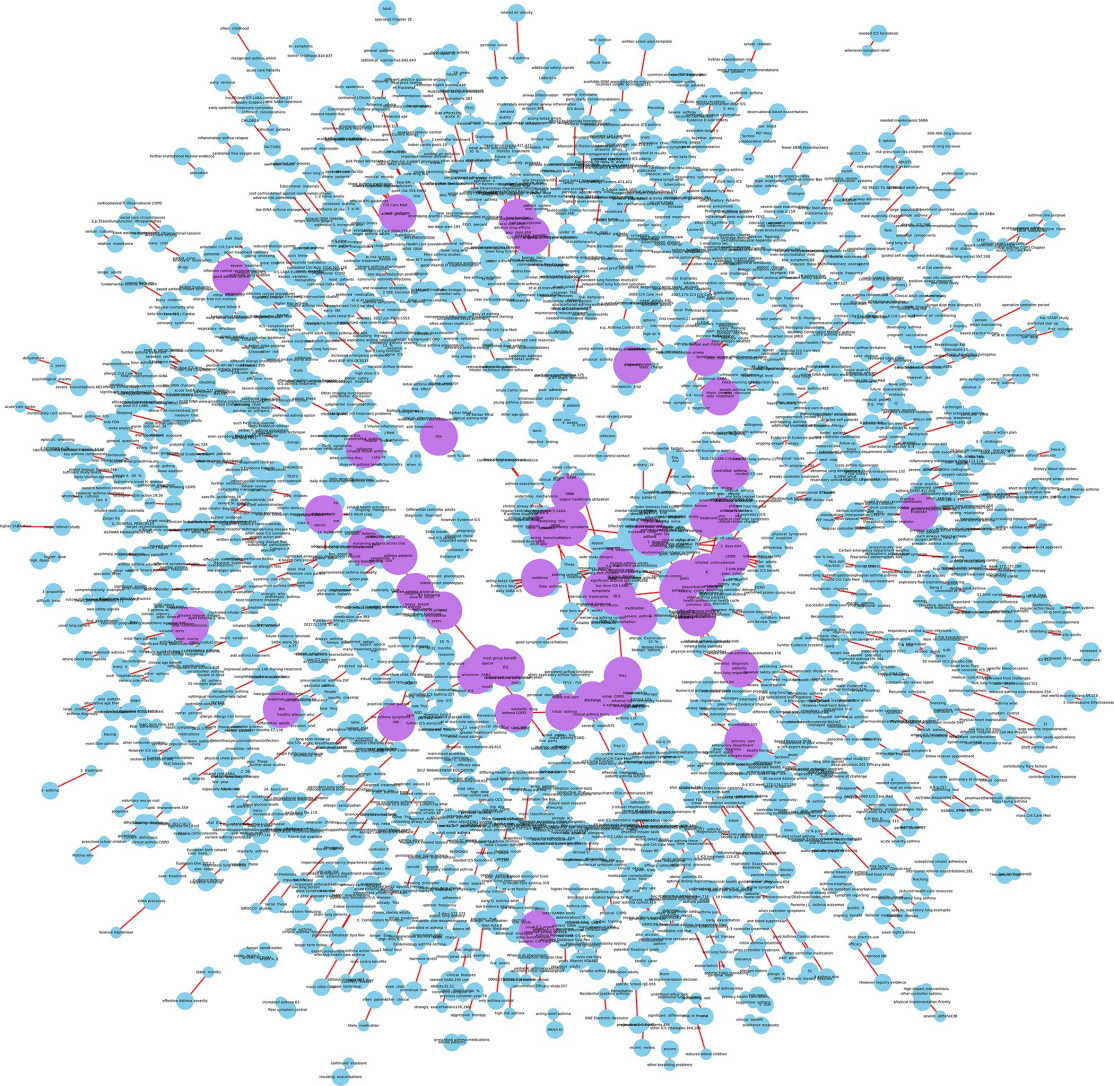
A joint-version KG is created by configuring constraints for multiple inputs related to a single entity.

Based on the basic KG, how to know the importance of entities? The higher importance of entities can be defined if the entity is not only used as the subject or object entity in the current triplet, but also this entity is used as the subject or the object inside the other triplets. This implies that the specific entity has multiple inputs and/or multiple outputs. Thus, we develop another version of KG called join-version KG. The overlapped entities will be colored and shown in the joint-version KGs in Fig 5.

In order to determine the importance of entities in the basic KG, we created a joint-version KG, which considers entities with multiple inputs and outputs as more important. The overlapping entities are shown in different colors in the joint-version KGs, with orange dots representing entities overlapping in two triplets, and purple dots representing entities overlapping in three triplets. In Fig 5, 41 purple dots were found. The constraint of having four subject entities and three object entities was set, resulting in 16 joint points found during Level Four processing.

The process of determining the significance of entities in the basic KG is complex. An entity is considered to be more important if it appears not only as a subject or object entity in the current triplet, but also as a subject or object in other triplets. This means that the entity has multiple inputs and outputs. To further understand this, we created a joint-version KG where the overlapped entities are colored and displayed. The importance of an entity is indicated by the number of overlapped instances, with joint points colored in orange and purple.

However, exploring all possible combinations of key entities with various inputs and outputs is not feasible. For example, there are seven combinations for the constraint ’three’, such as key (3,0), key (3,1), key (3,2), key (3,3), key (0,3), key (1,3), and key (2,3). For a constraint ’n’, there are 2*n+1 combinations. This method can help identify more important entities, but it is important to note that less frequently occurring keywords may still be crucial to the domain. To address this issue, we have introduced the interactive knowledge graph construction which provides an environment for researchers to explore and discover relevant entities in their field.

### 4. Interactive KG construction

**Fig 6:**
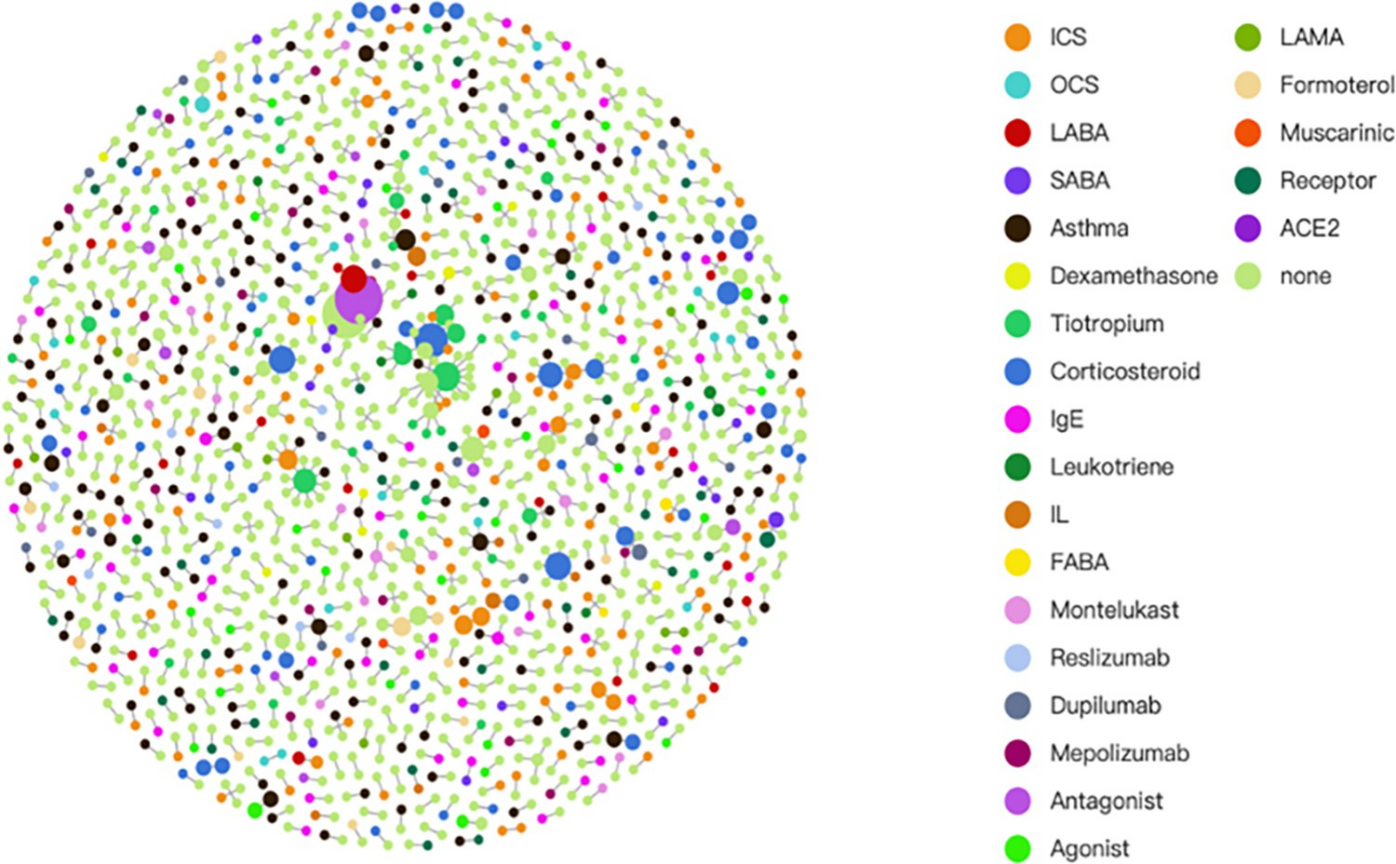
An interactive KG with entities on the right for exploration is created.

To collect important entities, an exhaustive search of all constraints can be done up to a certain number, but this can be time-consuming. Instead, we offer an interactive KG that allows users to explore entity relations on their own. Researchers can freely select desired entities and observe their interactive relationships without constraints. The interactive KG also has zoom-in and zoom-out features through a mouse sliding button, after the python scripts are converted to HTML files. Fig 6 shows the interactive KG environment, with entities displayed on the right and various colored dots to differentiate the entities [23].

**Fig 7:**
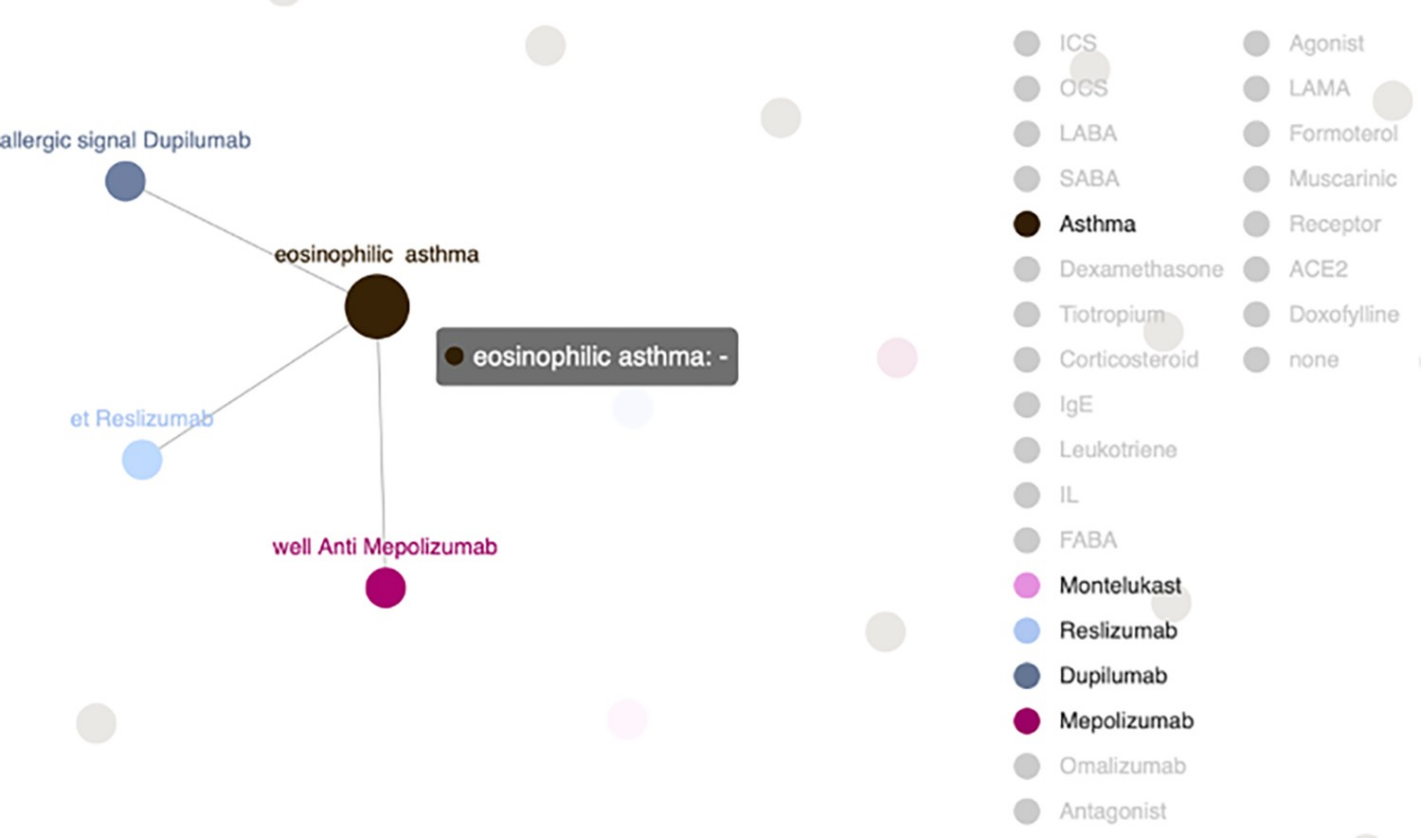
A sub-KG extracted from user-selected entities in an interactive KG.

For instance, users can select specific entities and examine their relationships. They can zoom in on the KG to see the relationships in detail. As an example, Fig 7 shows that the medications Dupilumab, Reslizumab, and Mepolizumab are related to asthma. This step enables researchers to understand existing knowledge. Although Dupilumab, Reslizumab, and Mepolizumab are not frequently used keywords, the interactive KG provides a platform for researchers to delve deeper and uncover sparse, unknown, but potentially crucial knowledge.

The interactive KG environment, as described above, is a valuable tool for researchers as it allows for self-exploration of relationships between entities. However, it’s also important to understand the specific keywords (relations) and entities that researchers are interested in. To address this, we have designed a method for extracting relationships and entities based on the accessed keywords, which is a process of knowledge distillation.

### 5. Knowledge distillation

In Level One processing, the VOSViewer tool is used to categorize articles. The resulting basic KG is complex and contains many irrelevant entities, so in Level Two processing, we use a method of sub-KGs to analyze and refine the entities and remove triplets with missing data. The joint-version KG enables us to identify important entities based on the number of inputs and outputs of the ’key’ entity. Professional researchers then validate these important entities in Level Three processing. In Level Four processing, an interactive KG is developed to allow researchers to explore relationships between important and sparse but crucial entities. The explored relations and entities are collected and distilled in response to user inquiries in Level Five processing.

## Results

**Fig 8:**
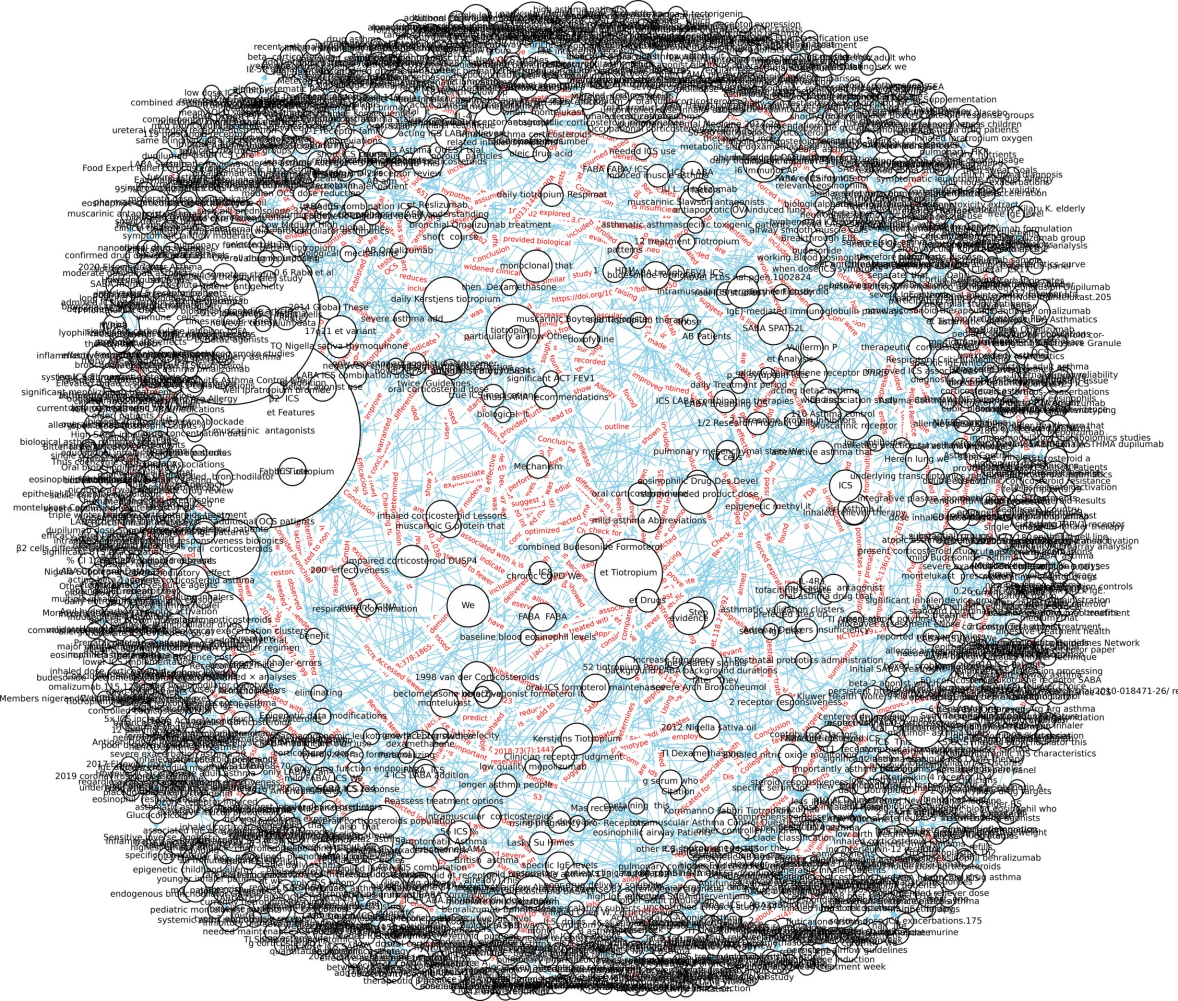
A validated medication KG is built.

**Fig 9:**
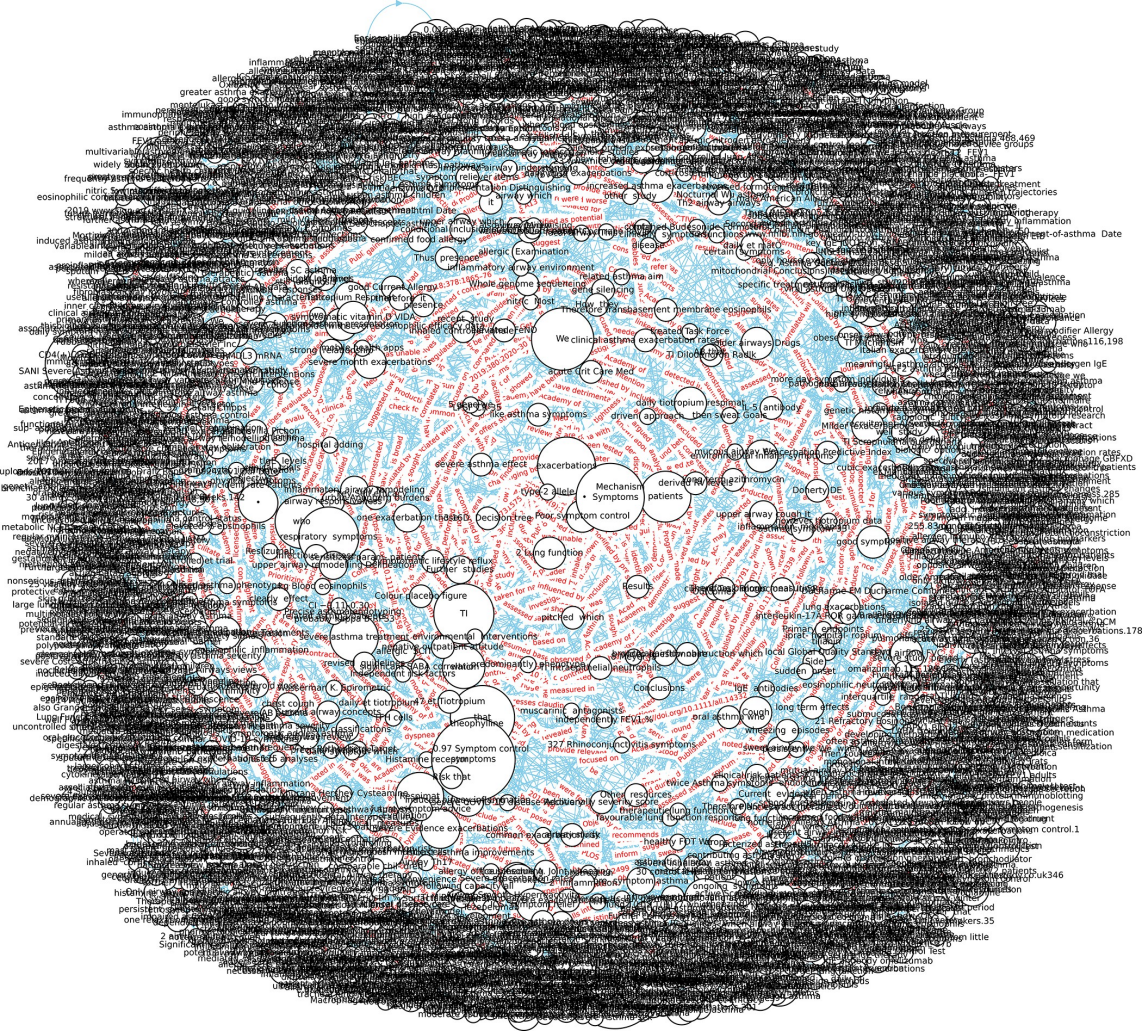
A validated symptom KG is built.

**Fig 10:**
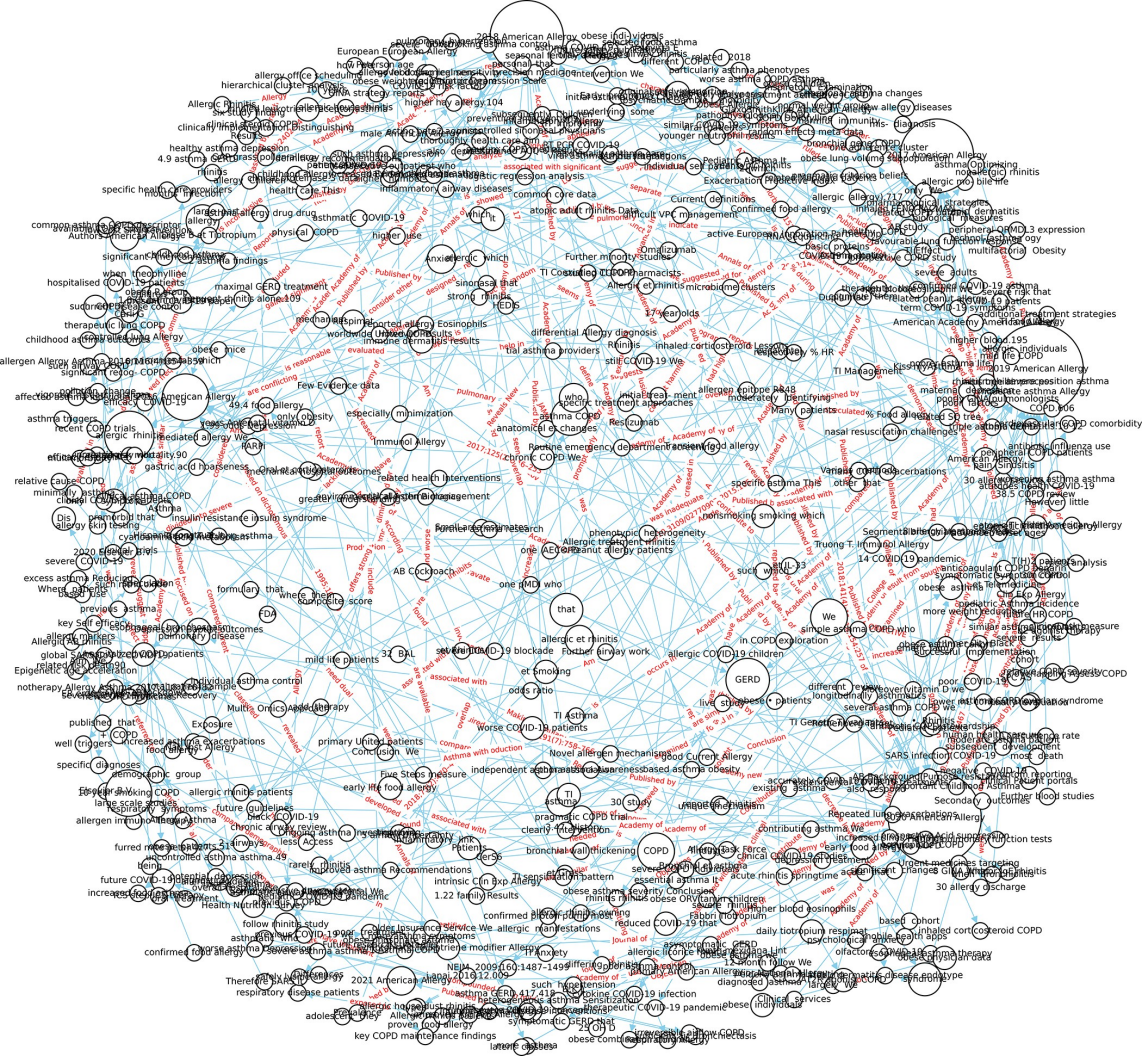
A validated comorbidity KG is built.

Constructing a KG can be challenging, as it requires collecting high-quality entities and relations. Traditional tools lack the flexibility to generate these entities effectively. In contrast, we employ MLR techniques in a 5-step process to generate high-quality triplets. Verification and validation are crucial in this process to ensure accuracy. Our final KGs include a medication KG, a symptom KG, and a comorbidity KG, as shown in Figs 8–10 respectively. We offer a flexible development environment for users who want to configure or redesign their desired KGs. The distilled relations and corresponding entities are available for inquiry.

**Fig 11:**
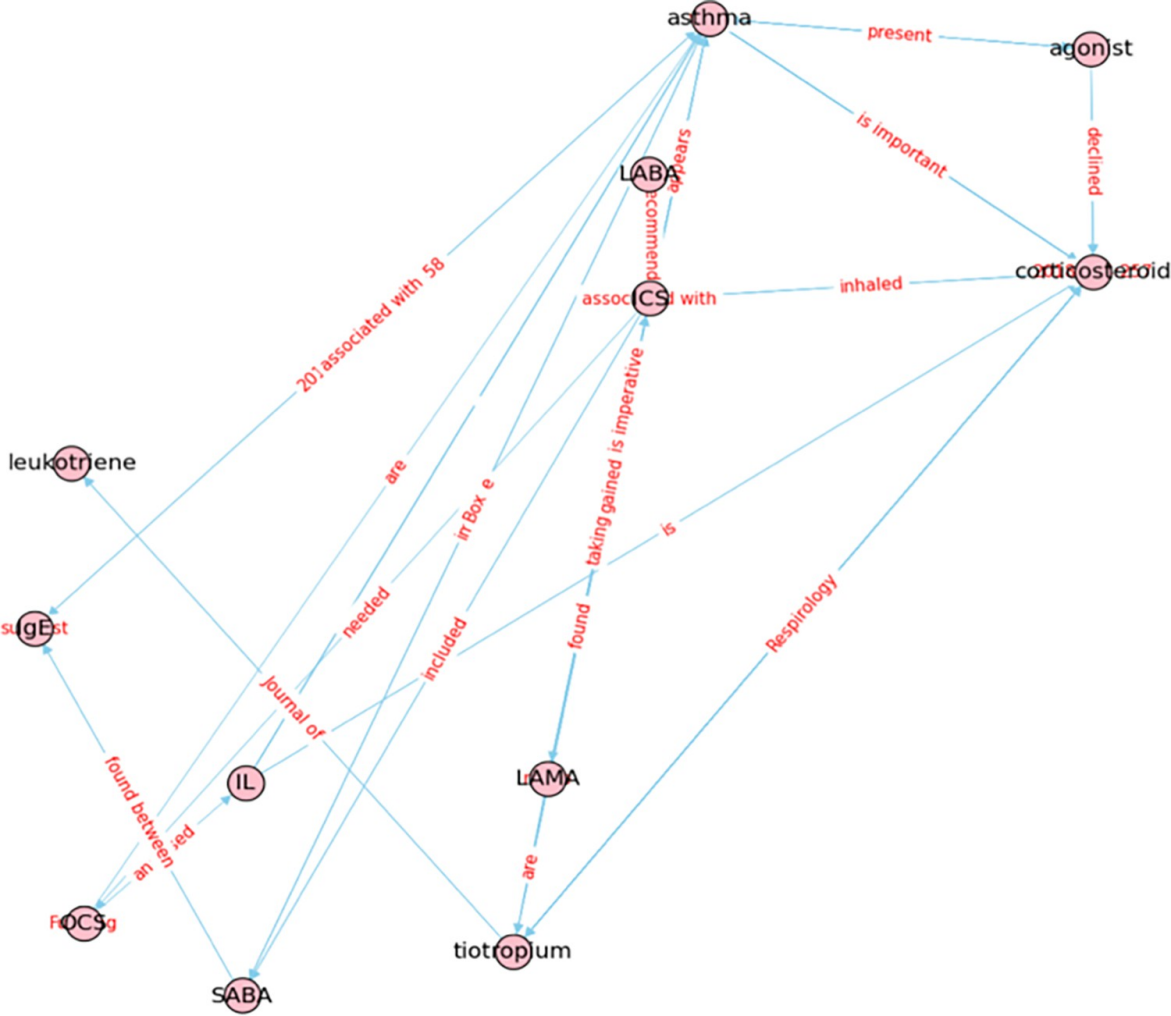
Knowledge distillation from a medication KG when users inquire about "asthma".

Fig 11 displays the knowledge distilled from a medication KG in response to the inquiry "asthma." The plot highlights the various treatments available for asthma patients. Asthma can be treated using inhaled corticosteroids (ICS) and/or oral corticosteroids (OCS). It is more effective if ICS is used in combination with short-acting +beta2-agonists (SABA) or long-acting beta2-agonists (LABA). For serious asthma cases, it is crucial for the patient to use ICS in combination with long-acting muscarinic antagonists (LAMA), with Tiotropium being the first available LAMA for asthma treatment. The use of Tiotropium can also provide relief by reducing Leukotrienes, which can cause asthma by inducing interleukins (IL). IL can be reduced by OCS and/or corticosteroid treatment, while Immunoglobulin E (IgE) that are antibodies produced by the immune system can be reduced by using SABA for asthma treatment.

**Fig 12:**
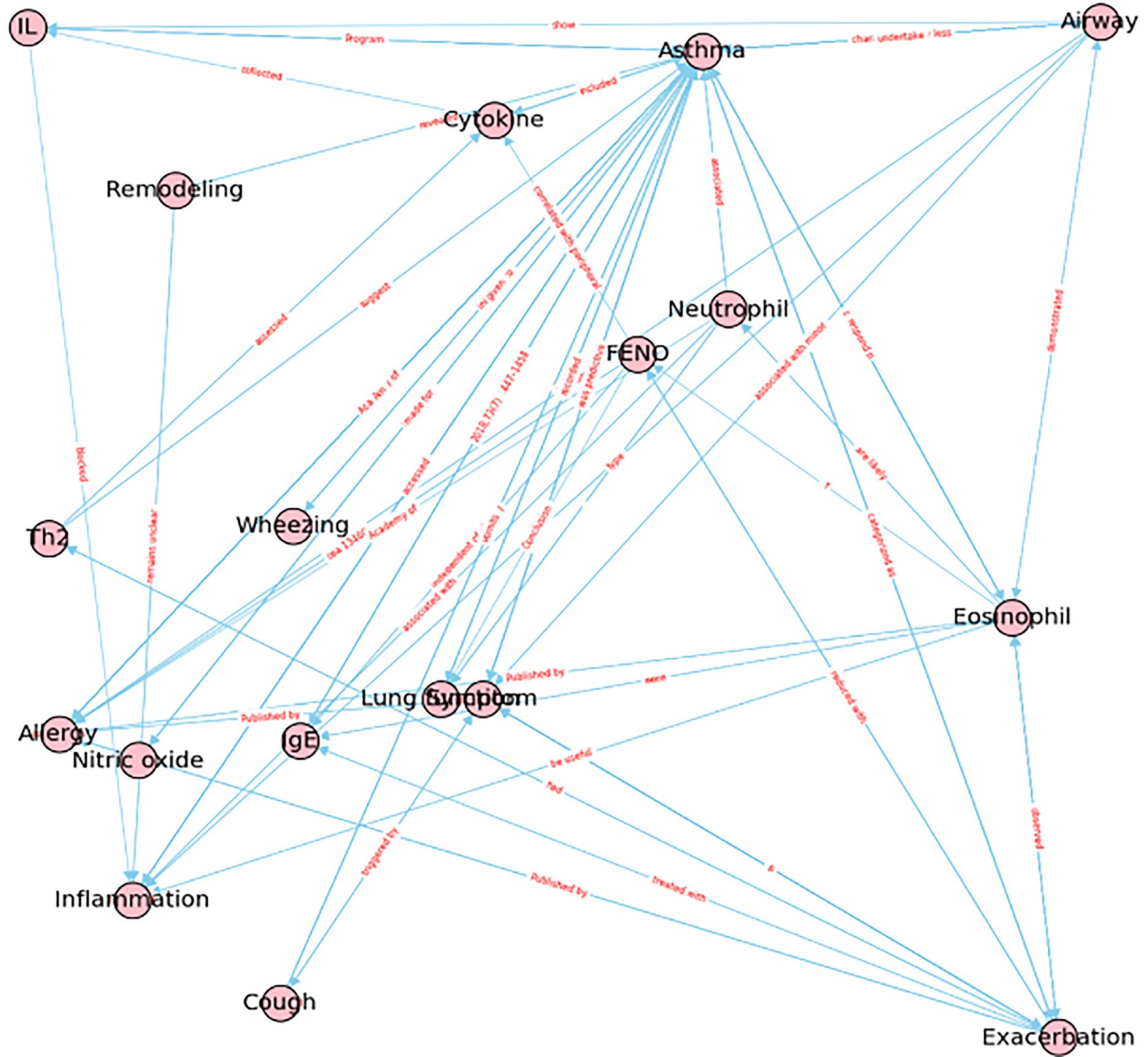
Knowledge distillation from a symptom KG when users inquire about "asthma".

The knowledge distillation results in Figs 11 and 12 provide a comprehensive understanding of asthma therapy guidelines, medication information, and symptoms respectively. Fig 11 displays the medication information for asthma patients, highlighting various treatments such as inhaled corticosteroid (ICS), oral corticosteroid (OCS), and combination therapies. Fig 12 shows the distillation from the symptom KG, showcasing symptoms such as wheezing, coughing, airway inflammation, and airway remodeling. It highlights the underlying causes of these symptoms, such as eosinophils, infiltration of neutrophils, IgE, cytokines, and airway inflammation. The exhaled fraction nitric oxide (FeNO) level is also discussed as a biomarker of T-helper cell type 2 (Th2) airway inflammation, and the study aimed to determine its use in differentiating between patients with controlled, partially controlled, and uncontrolled asthma.

**Fig 13:**
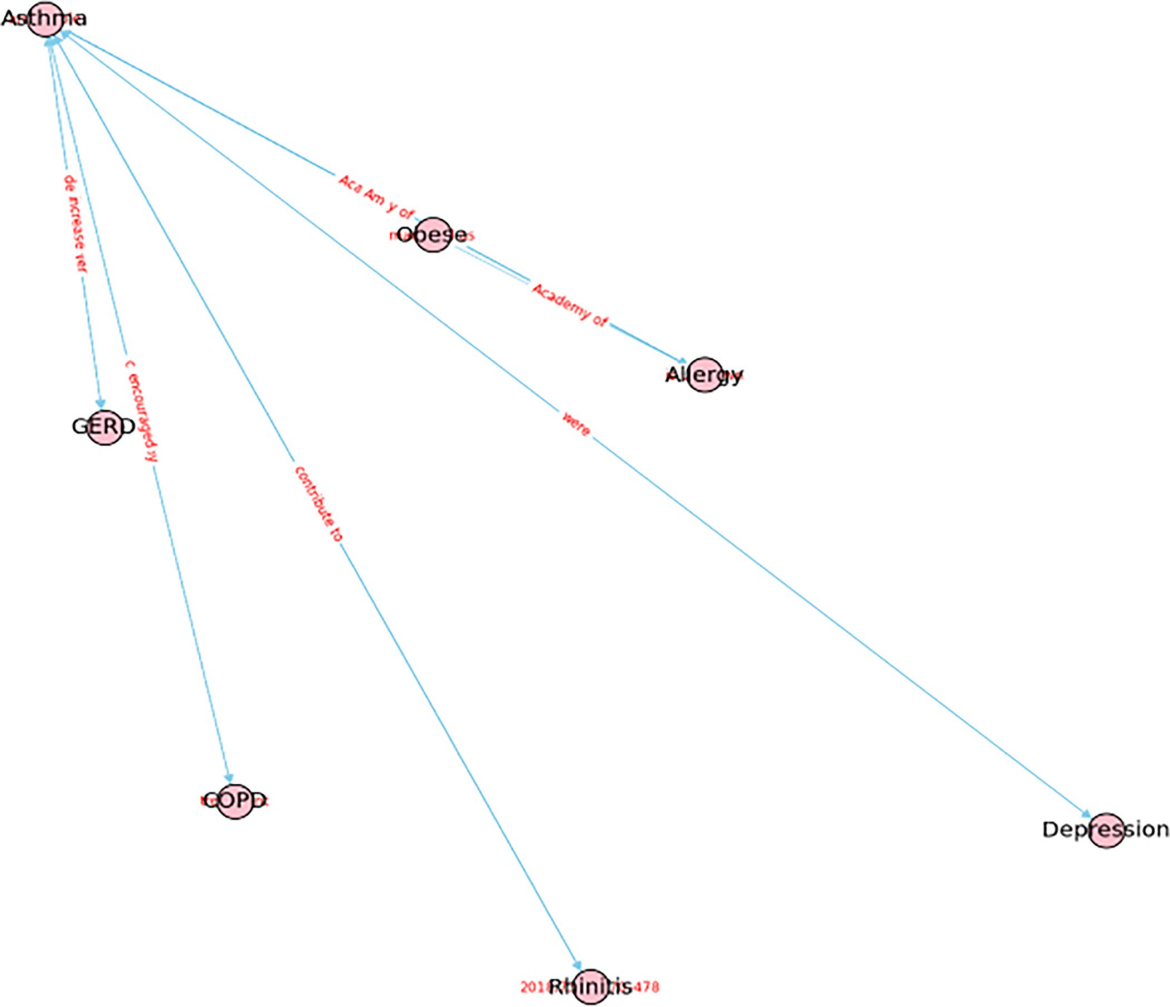
Knowledge distillation from a comorbidity KG when users inquire about "asthma".

Fig 13 illustrates the comorbidities associated with asthma that are distilled from the comorbidity KG. Asthma is associated with several conditions including allergies, obesity, depression, Chronic Obstructive Pulmonary Disease (COPD), Gastroesophageal reflux disease (GERD), and rhinitis. These results are generated through the application of our knowledge distillation techniques on the final three KGs covering Medication, Symptom, and Comorbidity.

## Discussion

The aim of this research is to enhance the construction of KGs through improved methods and the creation of basic, joint, and interactive versions. KGs are complex semantic networks that consist of entities, concepts, and relationships, making it challenging to accurately represent professional entities and relationships in medical KGs. To tackle this issue, we utilized machine learning to analyze thousands of articles, automatically identifying disease-related entities and relationships, and creating a verified database. Additionally, we developed an interactive exploration tool to discover connections among entities and distilled the knowledge networks for user searches. Although we processed a large volume of articles, there are still limitations in accessing high-quality papers and journals.

## Limitations

Constructing a knowledge graph presents several advantages, yet it also entails certain drawbacks. The process is resource-intensive, demanding substantial time, effort, and expertise for both development and maintenance. Maintaining data quality and accuracy poses a significant challenge within the knowledge graph, given that inaccuracies or outdated information can compromise its effectiveness. Striking a balance between establishing detailed relationships and avoiding overwhelming complexity is crucial. Excessively intricate knowledge graphs may pose challenges in terms of management and interpretation. Finding the right equilibrium is essential to maximize the benefits of a knowledge graph while mitigating potential challenges.

## Conclusions

The use of KGs in healthcare and medical fields has gained increasing attention in recent years, but a systematic methodology to build a professional KG is still lacking. To address this, we use VOSViewer to categorize papers and articles into different types of KGs such as medication, symptom, and comorbidity, which we refer to as Level One refinement. Then, we create a basic comprehensive KG and sub-KG of the top 15 relations, collecting the related entities for further refinement in multiple levels. These collected triplets must be verified, validated, and approved by medical professionals and researchers, with only high-quality triplets being stored in our database, known as Level Three refinement. To find the inputs and outputs of entity linking, we develop a scheme and identify entities with multiple inputs and outputs, called joints, for which we develop a novel joint-version KG. At the same time, we create an interactive KG to allow medical professionals to explore related entities and treatments or diseases, which is called Level Four refinement. Finally, we develop an ask and solution portion using our innovative knowledge distillation techniques, referred to as Level Five refinement. Additionally, a system to automatically read multiple files within a folder is also required. Our work contributes to the field by providing a comprehensive design flow that focuses not only on building KGs but also on maintaining the quality of entities. The final result will be a complete solution, with distilled knowledge and entities and relations plotted based on a search keyword.

To start, we use VOSViewer to categorize articles into different types such as asthma medication, symptoms, and comorbidity. We apply NLP techniques, including tokenization, part-of-speech tagging, parsing, lemmatization, and named entity recognition, to perform machine learning and generate triplet tables from the categorized articles. These tables are then combined into a comprehensive table ready for KG plotting. Using the networkx package in the Python library, we construct a KG based on the complete triplet table and generate sub-KGs with the top 10 verbs (known as relations). The related entities are collected and used as a foundation for our database.

However, some triplets in the table may have complex relationships, leading to overlapping subject and/or object entities, which we refer to as joint entities. These joint entities are marked in a special color within the KG plot and are further validated by medical professionals and researchers. As it is not possible to exhaustively search for all possible combinations of joint entities, we developed an interactive KG framework for researchers to explore various possibilities for these entities. This exploration may lead to the discovery of rare and crucial entities, resulting in a high-quality data center.

Our design approach follows a step-by-step process, following a top-down methodology. At each step, we address the issues from the previous stage and develop the KG and corresponding methodologies. The focus is not just on building the KG, but also maintaining the quality of the entities. The final outcome is a comprehensive KG that can be visualized, and its knowledge distilled based on the user’s search keyword, providing a complete solution.

### A chatbot system design

Our chatbot system is designed to leverage our proprietary KG structure, enabling users to ask questions accurately and receive quick and precise answers. To enhance the accuracy of our question-answering system, we prioritize the entity relations based on their frequency. By predicting the likely triple combinations that users may inquire about, our system can provide accurate responses. To measure the performance of our system, we employ performance metrics such as recall, precision, F1, MRR (Mean Reciprocal Rank), MAP (Mean Average Precision), and nDCG(normalized Discounted Cumulative Gain). These metrics allow us to assess the system’s ability to deliver precise and reliable information. Our ultimate goal is to ensure that our chatbot system excels in both accuracy and speed, enabling users to engage in effective and seamless conversations.

We utilized the 2022 version of GINA as our KG for the purpose of illustration. The processing of the data yielded a total of 13836 triplets. Subsequently, the previous extraction keywords were refined to produce 7688 triplets, and the incomplete triplets were removed, resulting in a final set of 860 triplets. Overlapping triplets were further eliminated, leaving us with a set of 202 triplets.

**Fig 14:**
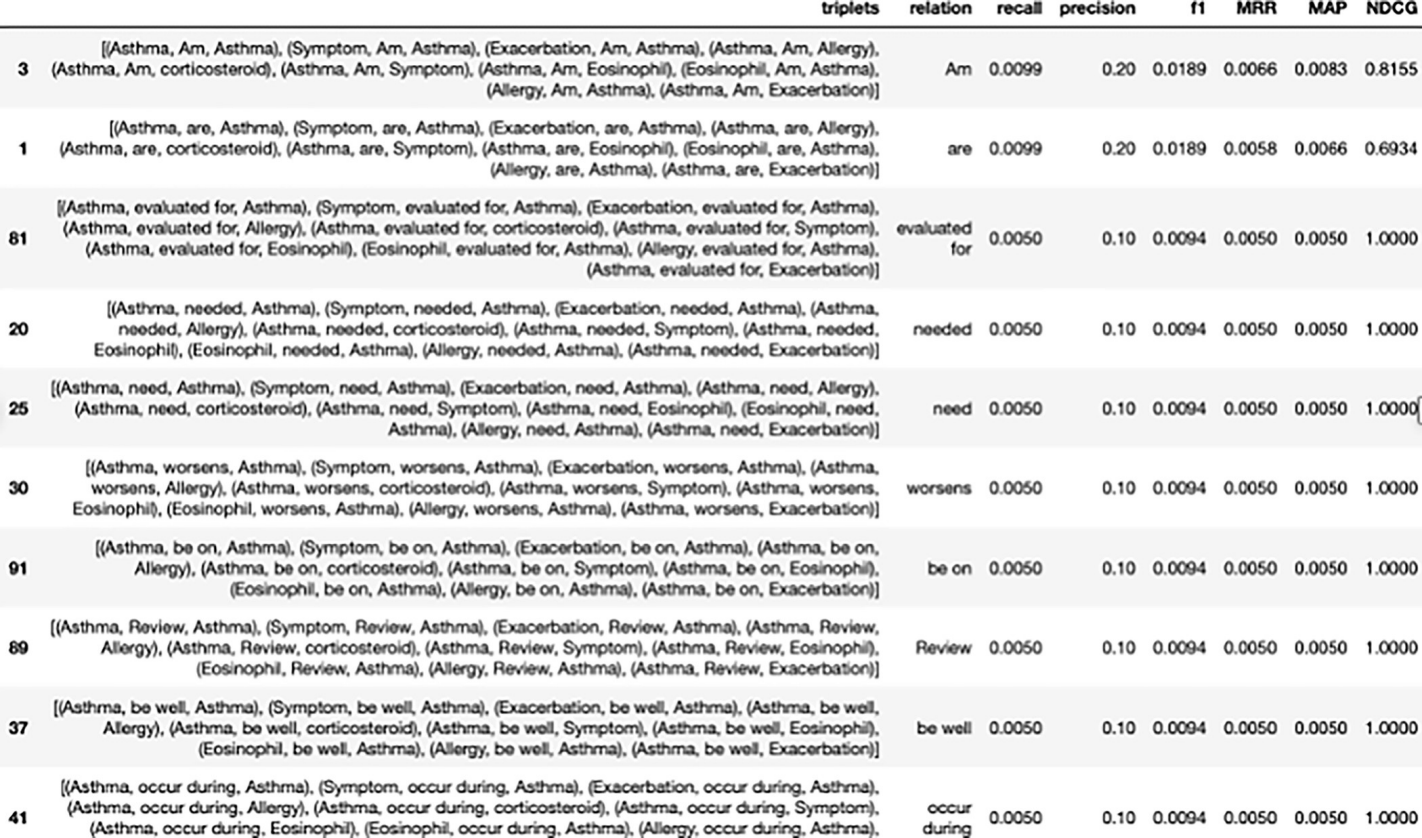
Performance metrics for triplet alignment.

To enhance query efficiency, we tested 202 triplets, also known as ground truth triplets, for recall and precision by merging triplets. We assumed that the intersection of 10 sets of predicted triplets above and 202 ground truth triplets resulted in two common triplets: (’Asthma’, ’Am’, ’Asthma’) and (’Exacerbation’, ’Am’, ’Asthma’). The overall hit rate, or recall, was calculated as 2 out of 202, resulting in a recall of 0.0099. The accuracy rate, or precision, was calculated as 2 out of 10, resulting in a precision of 0.2, illustrated at the above table in Fig 14.

**Fig 15:**
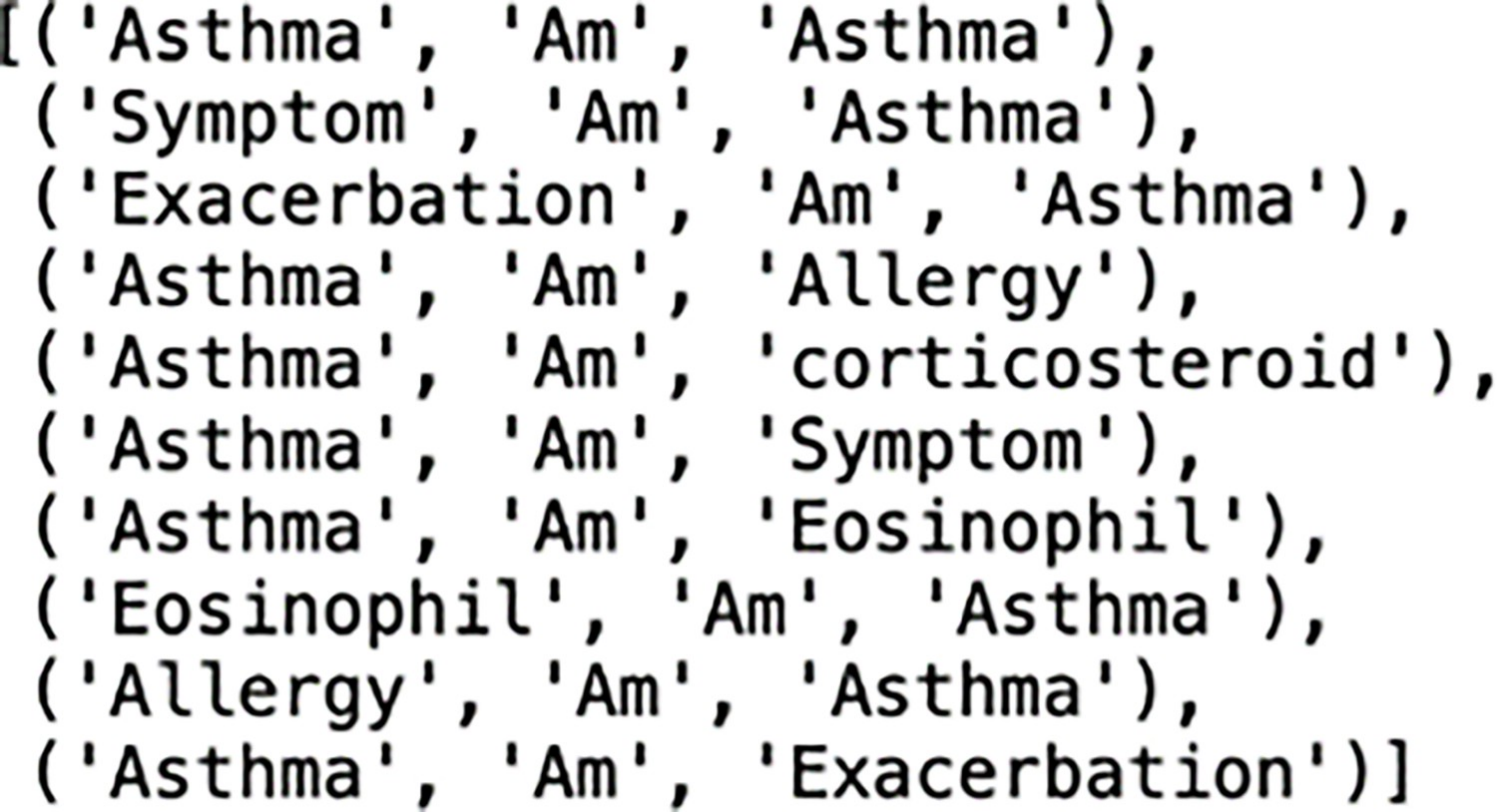
Considering the utilization of 10 query triplets.

As an example, above for Row One, we identified the top 10 most common entity pairs, added a relation to each pair such as ‘*Am’*, and created 10 sets of query triplets, referred to as predicted triplets listed I Fig 15.

The MRR is a metric used to evaluate the effectiveness of a ranking algorithm by measuring the average reciprocal rank of the correct answers. To calculate the MRR, we first need to compute the cumulative score by summing the reciprocal of the ranks of all the correct answers. Once we have the cumulative score, we can divide it by the total number of ground truth triplets to obtain the MRR. For example, if the first and third groups are the correct answers such as (’Asthma’, ’Am’, ’Asthma’) and (’Exacerbation’, ’Am’, ’Asthma’), the cumulative score would be 1/1+1/3 = 4/3; and if there are 202 ground truth triplets, the MRR would be 0.0066.

The Mean Average Precision (MAP) is a popular metric for evaluating the performance of information retrieval systems, particularly in the context of ranked retrieval. To compute the MAP, we first need to calculate the cumulative score by multiplying the reciprocal of the ranks of all the correct answers by their respective relevancy values and summing these values. Once we have the cumulative score, we can divide it by the total number of ground truth triplets to obtain the MAP. For example, if the first and third groups are the correct answers, and the first group is more relevant than the third group, the cumulative score would be (1/1) *1 + (1/3) *2 = 1/1 + 2/3 = 5/3, and if there are 202 ground truth triplets, the MAP would be 0.0083.

The provided example demonstrates how to calculate the Normalized Discounted Cumulative Gain (nDCG) for a set of retrieved items. In this example, the first and third groups were deemed to be relevant, while the remaining groups were not. The Discounted Cumulative Gain (DCG) was calculated by assigning a relevance score of 1 to the first and third groups and 0 to the remaining groups, and then summing these scores according to the DCG formula. The result was 1 + 1/log2(3) = 1.6313. The Ideal Discounted Cumulative Gain (IDCG) was calculated by sorting the relevance scores in descending order and calculating the DCG using the same formula. The result was 1 + 1/log2(2) = 2. Finally, the nDCG was calculated by dividing the DCG by the IDCG, which gave a result of 0.8155. This means that the ranking algorithm was able to retrieve a set of relevant items with a score that is 81.55% of the ideal score.

The example above serves as an illustration of how performance metrics can be utilized to showcase the quality of our system, enabling us to achieve fast and accurate answers. By leveraging these metrics, we can effectively demonstrate the system’s capabilities and its ability to provide prompt and precise responses. Our primary objective is to consistently enhance the system’s quality, ensuring that users receive quick and accurate information during their interactions. We are dedicated to maintaining a high standard of excellence in our chatbot system, offering a seamless user experience that meets and exceeds expectations.

## Supporting information

We have incorporated supplementary information files to visually represent our input, design, and output, with these associated files presented accordingly. The PDF document of the ’Global Initiative for Asthma (GINA) guideline article is transformed into a TXT file for use as the input (gina.txt). Additionally, we have developed Python code for knowledge graph construction, using the ’gina.txt’ file as an illustrative example (kg_construction_r2.ipynb). To optimize performance, we introduce key performance metrics, including MRR, MAP, NDCG, F1, and precision, for evaluating the impact of rearranging triplets in the triple tables (myfile_performance_one.csv). During the knowledge graph construction process, it is imperative to eliminate any missing data from the triplet tables to enhance the accuracy and precision of the resulting knowledge graph (myfile_cleaned_kg.csv).

S1 File (TXT)

S2 File (IPYNB)

S1 Table (CSV)

S2 Table (CSV)
