# Correction: A novel Multi-Level Refined (MLR) knowledge graph design and chatbot system for healthcare applications

**DOI:** 10.1371/journal.pone.0302620

**Published:** 2024-04-19

**Authors:** Huei-Chia Hsueh, Shuo-Chen Chien, Chih-Wei Huang, Hsuan-Chia Yang, Usman Iqbal, Li-Fong Lin, Wen-Shan Jian

An additional affiliation is missing for the fifth author. Usman Iqbal is also affiliated with School of Population Health, Faculty of Medicine and Health, University of New South Wales (UNSW), Sydney, NSW, Australia.
